# Rational In Situ Fabrication of ZnMoO_4_ Shielding Layers to Mitigate Zinc Degradation and Extend Battery Lifespan

**DOI:** 10.3390/mi17080982

**Published:** 2026-08-20

**Authors:** Xiaodong Zhang, Yan Zhang, Yingbin Liu, Kai Li, Changdong Chen

**Affiliations:** 1 School of Materials Science and Engineering, Pingdingshan University, Pingdingshan 467000, China; 2 State Key Laboratory of Space Power-Sources, Harbin Institute of Technology, Harbin 150001, China; zhangyhit@hit.edu.cn

**Keywords:** zinc-ion batteries, one-step immersion, Zn dendrite suppression, hydrogen evolution inhibition

## Abstract

Aqueous zinc-ion batteries (AZIBs) have garnered extensive attention owing to their high theoretical capacity, cost-effectiveness, and intrinsic safety. However, the practical deployment of AZIBs is severely hindered by deleterious side reactions, including surface corrosion, hydrogen evolution, and uncontrollable dendrite growth on the metallic Zn anode. In this work, we propose a simple one-step immersion strategy to in situ construct a ZnMoO_4_ (ZMO) protective coating on the Zn electrode. Mechanistically, the ZMO layer with polar surfaces exhibits a preferential adsorption affinity towards water molecules and Zn^2+^ ions. This synergistic adsorption behavior serves a dual function: it effectively excludes active water from the electrode surface to suppress hydrogen evolution, and simultaneously, the strong interaction with Zn^2+^ lowers the desolvation energy barrier, facilitating rapid Zn^2+^ desolvation at the interface. Furthermore, the resulting ZMO coating promotes a homogenized surface electric field and provides abundant nucleation sites, thereby guiding uniform Zn deposition and effectively mitigating dendrite formation. Consequently, the ZMO-modified Zn anode delivers significantly enhanced electrochemical reversibility and long-term cycling stability. This work provides a cost-effective and industrially viable surface engineering strategy to tackle the fundamental challenges of Zn anodes, paving the way for the commercialization of high-performance AZIBs.

## 1. Introduction

The continuous growth in energy demand, driven by societal advancement, has underscored the urgent need to address environmental pollution. Consequently, the global search for new clean energy sources has intensified [1,2,3]. The advent of lithium-ion batteries (LIBs) represents a significant milestone in energy storage systems [4]. Despite the successful large-scale application of LIBs, several challenges persist, including limited resources, high costs, and safety concerns about organic electrolytes and metallic lithium. These issues have notably constrained the further development of LIBs [5,6,7]. In contrast, aqueous energy storage devices have emerged as promising alternatives. Various aqueous batteries employing Zn^2+^, K^+^, Al^3+^, Ca^2+^, and other charge carriers have been developed [8,9,10,11]. Among these, AZIBs have gained attention from the favorable (electro)chemical stability of Zn metal. Additionally, Zn metal provides two-electron transfer with high volume capacity, low electrochemical potential, and plentiful natural abundance [12,13,14]. Thus, AZIBs are poised to become the next generation of secondary batteries.

Nevertheless, the progress of AZIBs faces major obstacles, including dendrite growth, passivation, corrosion, and HER. These reactions result in low CE and poor reversibility, and their interrelated nature can lead to capacity degradation and battery failure, severely impacting the electrochemical stability of AZIBs [15,16,17]. The irreversibility of the Zn anode stems from the unstable interface, which precipitates these side reactions. Therefore, optimizing the Zn/electrolyte interface and regulating the Zn^2+^ solvation structure plays a key role in boosting the Zn anode’s reversibility. New, numerous approaches have been adopted to tackle these problems, including structural design, interface modification, alloy construction, and electrolyte engineering [18,19,20,21,22]. While these measures can mitigate side effects to some extent, they also present limitations. Among these strategies, the construction of an artificial inorganic interface layer on the Zn anode has proven effective in suppressing dendrite formation and corrosion. For instance, nano-CaCO_3_, TiO_2_ nanolayers, and ZrO_2_ layers have been applied to the Zn surface [23,24,25]. The physical barrier provided by these coatings can effectively guide Zn^2+^ diffusion, thus extending the cycle life of the Zn anode. Nonetheless, most coatings are applied using slurry blade coating methods, which involve cumbersome processes with toxic organic solvents and may result in weak adhesion, inhomogeneity, and voids between the coating and Zn foil. Therefore, constructing a sustainable interface layer through in situ growth is highly desirable. Li et al. demonstrated the construction of a Zn_3_(PO_4_)_2_·4H_2_O (ZnPO) layer on the Zn foil surface by a hydrothermal method. As a zinc ion conductor, ZnPO facilitates the uniform deposition of Zn^2+^ and inhibits creation of Zn dendrites [26]. Similarly, Lu et al. in situ generated a zeolitic imidazolate framework-8 (ZIF-8) on the Zn surface through a hydrothermal process [27]. However, these strategies are complex, costly, and difficult to implement in practical applications. Therefore, identifying simple and cost-effective methods remains essential.

In recent years, ZnMoO_4_ has been explored as a functional inorganic coating material for Zn anodes. However, the currently reported methods for preparing ZnMoO_4_ coatings mainly rely on hydrothermal/solvothermal methods or multi-step synthesis processes, which have inherent limitations such as strict reaction conditions, cumbersome steps, high energy consumption, and the fact that they are not conducive to large-scale production. In this study, a layer of ZnMoO_4_ was in situ constructed onto the Zn anode surface (Zn@ZMO) by a simple soaking process. On this basis, we clearly explained the core innovation of the one-step room-temperature immersion method in this work while maintaining the unity of coating functionality (Figure S1). ZMO effectively keeps the Zn anode physically separated from the electrolyte, reducing the occurrence of parasitic reactions. The hydrophilic nature of the ZMO layer facilitates Zn^2+^ desolvation, facilitating homogeneous Zn^2+^ electrodeposition and restraining the evolution of Zn dendrites. Additionally, the ZMO significantly reduces battery polarization voltage, thereby extending battery life. Owing to the protective effect of ZMO, the CE of the Zn@ZMO//Cu half-cell reached 97.0% over 2000 cycles at 5 mA cm^−2^. The symmetrical Zn@ZMO//Zn@ZMO battery demonstrated a cycle life of 1000 h at 5 mA cm^−2^ with 1 mAh cm^−2^, while the Zn@ZMO//I_2_ battery maintained stability for over 10,000 cycles at 5 A g^−1^. Offering a fresh perspective, this straightforward and powerful modification method holds promise for the onward evolution of Zn anodes.

## 2. Results and Discussion

**Figure 1:**
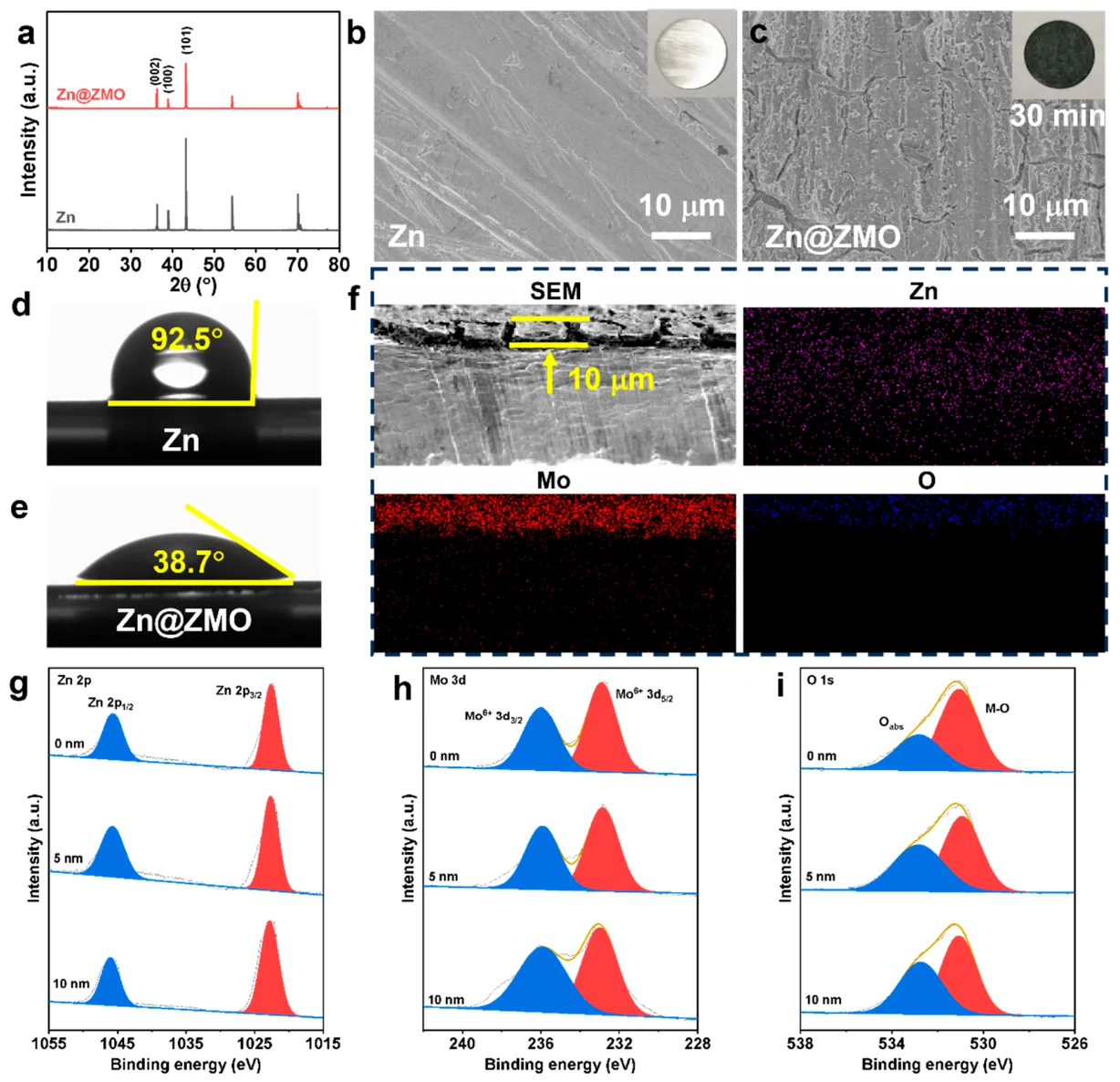
(**a**) XRD patterns, SEM images, and contact angles with electrolytes of (**b**,**d**) Zn and (**c**,**e**) Zn@ZMO. (**f**) Elemental mappings and cross-sectional SEM image of Zn@ZMO. XPS spectral regions for (**g**) Zn 2p, (**h**) Mo 3d, and (**i**) O 1s at distinct Ar sputtering depths on the Zn@ZMO anode. The insets of (**b**,**c**) present the optical photographs of Zn and Zn@ZMO, respectively.

The addition of phosphomolybdate solution to the Zn foil surface rapidly transformed the yellow solution into a deep blue color. After the mixture was left to stand for 30 min and the surface solution was then rinsed away, a distinct film appeared on the Zn foil (Figure S2). X-ray diffraction (XRD) analysis revealed that the three main characteristic peaks of Zn@ZMO corresponded to the (002), (100), and (101) planes of Zn metal, with no other significant peaks (Figure 1a). Scanning electron microscope (SEM) images showed that the pristine Zn had a flat surface (Figure 1b), whereas Zn@ZMO exhibited a dense protective layer (Figure 1c). As illustrated in Figure 1f, a protective layer approximately 10 µm thick was formed on the Zn foil surface, with Zn, Mo, and O elements uniformly distributed. The contact angle of the electrolyte on pristine Zn (92.5°) was significantly larger compared with that on Zn@ZMO (38.7°) (Figure 1d,e), indicating that the ZMO protective layer possesses hydrophilic MoO_4_^2−^.

Furthermore, the modified Zn anode was subjected to X-ray photoelectron spectroscopy (XPS) etching analysis as a function of sputtering depth, revealing that the surface substances were relatively stable and uniform, consisting mainly of Zn^2+^, Mo^6+^, and metal–oxygen bonds. The Zn 2p spectrum displayed four peaks at 1047.0, 1045.2, 1023.6 and 1022.1 eV, corresponding to Zn^2+^ 2p_1/2_, Zn^0^ 2p_1/2_, Zn^2+^ 2p_3/2_ and Zn^0^ 2p_3/2_, respectively (Figure 1g) [28]. In the Mo 3d spectrum, the two peaks were identified as Mo^6+^ 3d_3/2_ and 3d_5/2_ (235.8 and 233.1 eV, respectively) (Figure 1h) [29].

Two peaks at 530.4 eV and 531.8 eV were observed in the O 1s spectrum, corresponding to metal–oxygen bonds and surface-adsorbed oxygen, respectively (Figure 1i) [30]. The Zn foil was soaked in the phosphomolybdate solution for an extended period of 48 h to further investigate the component of the protective layer. The substance formed on the Zn foil surface was identified as ZnMoO_4_·0.8H_2_O (PDF#25-1025) (Figure S3). We conducted Raman characterization on Zn foil soaked for 30 min and 48 h. Two types of Zn foils exhibit the same characteristic peaks (MoO_4_ and ZnO_6_), but the characteristic peak intensity of the Zn foil soaked for 30 min is weaker, indicating that a ZnMoO_4_ protective layer can be formed on the surface of the Zn foil soaked for 30 min (Figure S4). A ZnMoO_4_ protective layer was in situ built on Zn foil upon the addition of phosphomolybdic acid. The absence of detectable ZnMoO_4_·0.8H_2_O in the XRD analysis of the Zn sheet soaked for 30 min can be attributed to the thinner protective layer and lower ZnMoO_4_·0.8H_2_O content generated during the shorter exposure time. Zn@ZMO with a thinner protective layer as the electrode is more conducive to the transport of Zn^2+^ ions.

**Figure 2:**
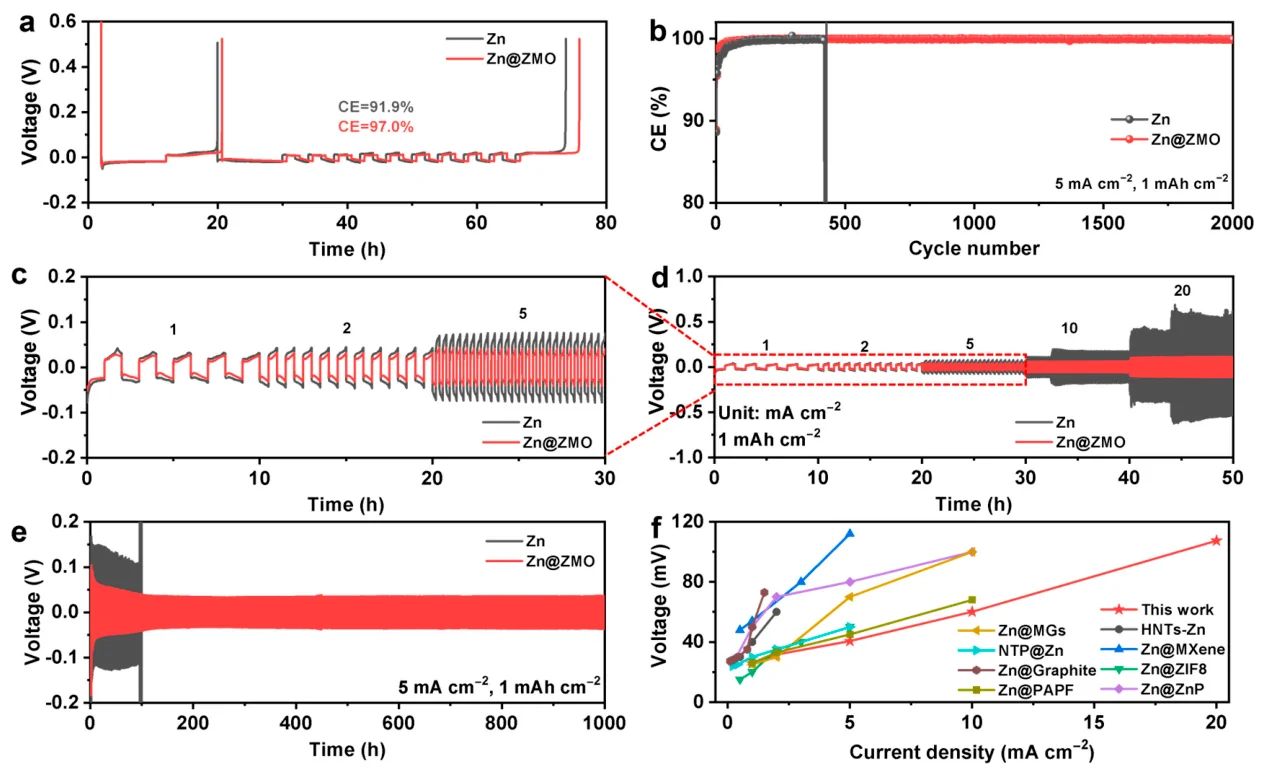
(**a**) Voltage profile for Zn//Cu and Zn@ZMO//Cu half-cells. (**b**) CE of Zn//Cu and Zn@ZMO//Cu batteries at 5 mA cm^−2^ with 1 mAh cm^−2^. (**c**,**d**) Rate performance of the Zn//Zn and Zn@ZMO//Zn@ZMO symmetrical batteries. (**e**) Cycle life of the Zn//Zn and Zn@ZMO//Zn@ZMO batteries at 5 mA cm^−2^. (**f**) Comparison of polarization voltage versus current density for the Zn@ZMO//Zn@ZMO battery and recently reported modified Zn anode.

The practical impact of the ZMO interlayer on Zn was evaluated by assembling a coin-type battery. In order to determine the optimal coating preparation time and optimize the reversibility of the Zn anode, we designed and implemented Zn anodes with different soaking times of 10, 30, and 60 min from the beginning (Figure S5). The CE of the Zn@ZMO//Cu half-cell reached 97.0%, as determined using the ‘reservoir half-cell’ method [31], which is superior to the CE of 91.9% observed for the bare Zn//Cu half-cell (Figure 2a). This enhancement indicates that the ZMO layer significantly improves Zn^2+^ deposition and stripping behavior, thereby enhancing the reversibility of the process. Additionally, the Zn@ZMO//Cu half-cell remained stable for 2000 cycles at 1 mAh cm^−2^ and 5 mA cm^−2^, whereas the Zn//Cu half-cell exhibited pronounced fluctuations after 400 cycles. This result demonstrates the ZMO layer’s effectiveness in suppressing side reactions (Figure 2b). Furthermore, Zn@ZMO//Zn@ZMO symmetric batteries were cycled in a stable manner for 1600 h (voltage hysteresis ~38.4 mV) and 1000 h (voltage hysteresis ~39.4 mV) at 1 mA cm^−2^ and 5 mA cm^−2^, respectively (Figure 2e and Figure S6).

Conversely, the Zn//Zn battery maintained stability for only 100–200 h, accompanied by a slightly higher voltage hysteresis of approximately 0.19 V (1.0 mA cm^−2^) and 0.22 V (5.0 mA cm^−2^). This degradation is attributed to continuous hydrogen generation during cycling, which results in poor electrode contact and eventual cell failure (Figure S6). The ZMO protective layer effectively mitigates hydrogen generation, thereby extending the battery’s lifespan. The Zn@ZMO//Zn@ZMO battery maintained a small voltage hysteresis and stable operation at 1 mAh cm^−2^ across different current densities, owing to the ZMO protective layer (Figure 2c,d). The polarization voltages for the Zn@ZMO//Zn@ZMO symmetric cell were 25.6, 31.6, 40.6, 60.1, and 107.4 mV at 1, 2, 5, 10, and 20 mA cm^−2^, respectively. These polarization voltages are lower than those observed for the Zn//Zn symmetric cell (32.6, 44.6, 76.3, 174, and 568 mV), suggesting faster Zn^2+^ transport kinetics in the ZMO protective layer (Figure S7). Compared with modified Zn anodes reported in other studies, the Zn@ZMO//Zn@ZMO symmetric battery in this work demonstrates superior rate performance as measured by polarization voltage (Figure 2f and Table S1).

**Figure 3:**
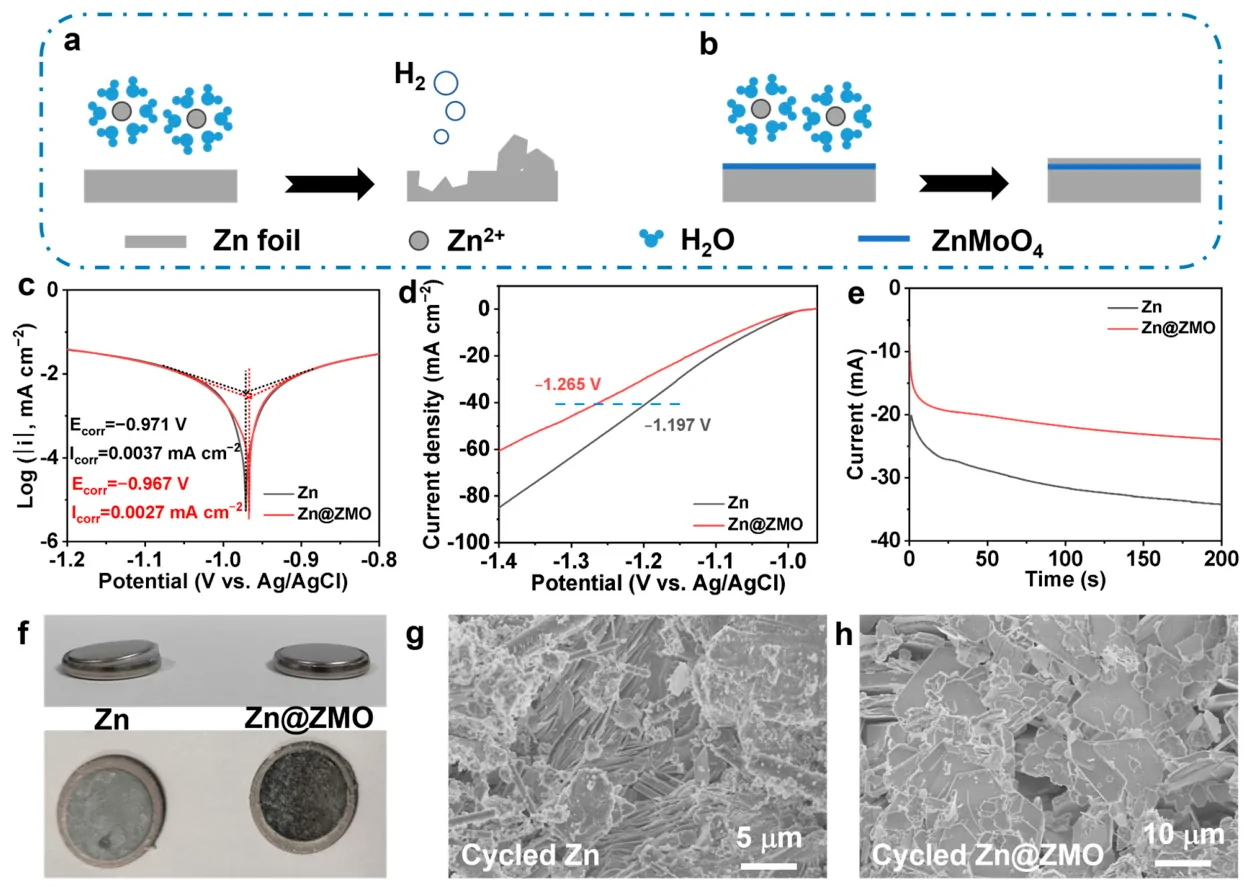
Schematic diagrams for Zn plating changes in (**a**) pristine Zn and (**b**) Zn@ZMO anodes. (**c**) Corrosion, (**d**) LSV, and (**e**) CA curves of pristine Zn and Zn@ZMO at 10 mV s^−1^. (**f**) Optical photographs of the cycled Zn//Zn and Zn@ZMO//Zn@ZMO symmetrical battery status. The SEM images of the cycled (**g**) Zn and (**h**) Zn@ZMO.

The Zn anode readily corrodes when exposed to aqueous electrolytes, leading to hydrogen evolution and dendrite formation (Figure 3a). Therefore, the Zn anode surface was modified in situ with a ZMO protective coating to effectively mitigate these parasitic reactions (Figure 3b). The efficacy of the ZMO protective layer was systematically validated through electrochemical tests. First, the corrosion potential (E_corr_) and corrosion current density (I_corr_) of Zn@ZMO, as measured by Tafel curves, were better (E_corr_ = −0.967 V and I_corr_ = 0.0027 mA cm^−2^) than those of pristine Zn (E_corr_ = −0.971 V and I_corr_ = 0.0037 mA cm^−2^), indicating the superior corrosion resistance conferred by the ZMO layer (Figure 3c). This superior anti-corrosion performance is attributed to the preferential adsorption of hydrophilic ZMO on the Zn surface, which effectively excludes aggressive H_2_O molecules and anions from direct contact with the underlying Zn substrate, thereby passivating the surface against corrosive attack. Second, linear sweep voltammetry (LSV) curves demonstrated that Zn@ZMO exhibits a more delayed hydrogen evolution potential (−1.265 V) compared to bare Zn (−1.197 V) at −40 mA cm^−2^ (Figure 3d). The same results were also observed in the Na_2_SO_4_ electrolyte (Figure S8). This suppression originates from the strong interaction between the ZMO and H_2_O molecules: the polar oxygen-rich surface of the ZMO-protected layer restricts free H_2_O activity via hydrogen-bonding interactions, while simultaneously, the preferential adsorption of Zn^2+^ onto the ZMO surface lowers the energy barrier for Zn Zn^2+^ desolvation, reducing the population of solvated H_2_O molecules available for reduction at the electrode/electrolyte interface [32].

These findings indicated that the ZMO layer effectively suppressed the HER. Additionally, chronoamperometry (CA) tests showed that the current fluctuation of Zn@ZMO remains more stable at a fixed overpotential of −150 mV during 200 s, demonstrating that the ZMO protective layer guides a 2D diffusion-controlled deposition mode and promotes lateral growth of Zn deposits, thereby effectively mitigating dendrite formation (Figure 3e). Comparison of the symmetric batteries after cycling revealed significant swelling in the Zn//Zn symmetric battery, attributable to excessive hydrogen production (Figure 3f). The cycled Zn anode shows noticeable corrosion, while the cycled Zn@ZMO anode remained relatively flat. SEM images further illustrated that the cycled Zn anode consisted mainly of irregular flake crystals, whereas the cycled Zn@ZMO anode was covered with flat hexagonal crystals (Figure 3g,h). Zn and Zn@ZMO sheets were deposited at 1 mA cm^−2^ for 5 h to compare morphological changes. From the XRD analysis of the plated Zn, the relative intensities of the (002) and (101) reflections (R = I_(002)_/I_(101)_) were found to vary. The XRD results indicated that Zn^2+^ preferentially deposited on the (101) plane of pristine Zn (R = 0.28), whereas the ZMO protective layer promoted Zn^2+^ to be deposited on the (002) plane (R = 0.58) (Figure S9a). SEM images confirmed that pristine Zn surfaces developed vertical dendritic structures (Figure S9b), while the Zn@ZMO surface remained flat (Figure S9c), consistent with the XRD results. Furthermore, soaking the electrodes in electrolyte demonstrated that the ZMO layer effectively inhibited the production of basic zinc salts (Figure S10). These outcomes emphasized how effectively the ZMO protective layer resolves associated issues such as HER and Zn dendrites in Zn anodes.

**Figure 4:**
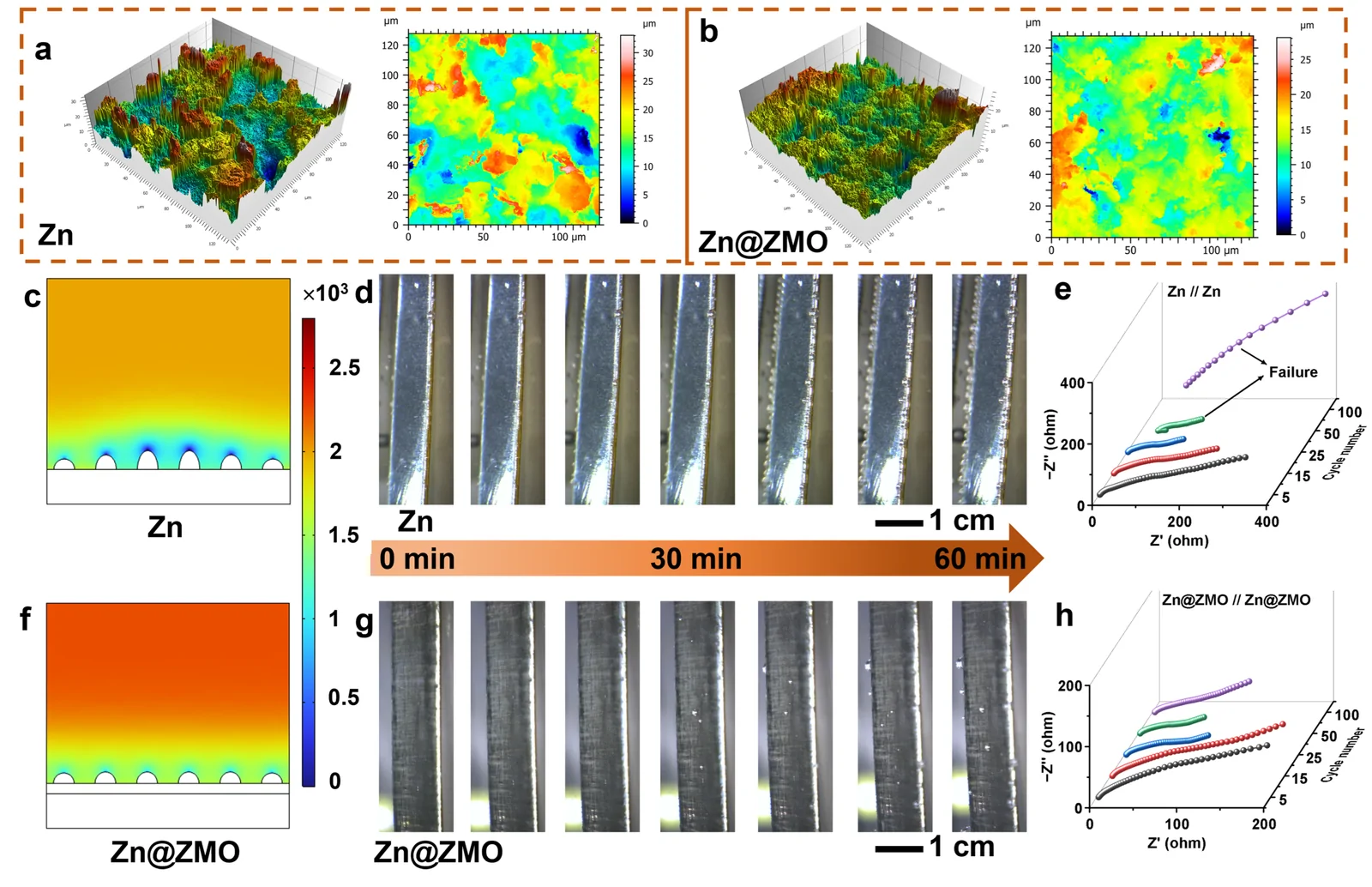
CLSM images of cycled (**a**) Zn and (**b**) Zn@ZMO anodes. Simulation results of the Zn^2+^ flux distribution on (**c**) pristine Zn and (**f**) Zn@ZMO. In situ low-resolution optical microscope of deposited Zn at 5 mA cm^−2^ onto (**d**) Zn and (**g**) Zn@ZMO. In situ EIS of (**e**) Zn//Zn and (**h**) Zn@ZMO//Zn@ZMO batteries.

For a more direct comparison between Zn and Zn@ZMO during the cycling process, a confocal laser scanning microscope (CLSM) was employed to measure the electrode surface flatness. The two-dimensional (2D) and three-dimensional (3D) CLSM images indicated that the cycled Zn surface is uneven, with surface roughness ranging from 5 to 30 µm (Figure 4a). By contrast, the cycled Zn@ZMO exhibits a comparatively smooth surface, with roughness primarily distributed between 5 and 15 µm (Figure 4b). The ZMO protective layer promoted uniform Zn^2+^ deposition and inhibited the creation of Zn dendrites. Further analysis of the Zn^2+^ flux distribution on Zn and Zn@ZMO was conducted employing COMSOL Multiphysics software. Zn^2+^ ions on the bare Zn anode favored accumulation at the tips, giving rise to an uneven ion distribution across the surface. (Figure 4c). These regions with notably enhanced Zn-ion flux accelerated Zn dendrite growth [33].

By contrast, the ZMO protective layer effectively redistributes and homogenizes Zn^2+^ ions, contributing to uniform Zn plating (Figure 4f). Additionally, in situ low-resolution optical microscopy was employed to directly observe changes around the Zn anode during charging. With increasing reaction time, a significant number of bubbles appeared on the bare Zn surface, indicating a strong HER (Figure 4d and Movie S1). Conversely, the Zn@ZMO surface showed no noticeable changes (Figure 4g and Movie S2), indicating that Zn@ZMO effectively mitigates HER during charging and discharging. XPS was employed to characterize the Zn@ZMO and verify the stability of the ZMO protective layer after cycling. The cycled Zn@ZMO electrode retained Zn^2+^, Mo^6+^, and metal–O species without significant changes in valence state, similar to the initial Zn@ZMO electrode (Figure S11). Furthermore, in situ EIS shows that the Zn//Zn battery exhibits fluctuations and even a significant increase in impedance after 50 cycles, indicating poor internal contact of the battery (Figure 4e). By contrast, the EIS of the Zn@ZMO//Zn@ZMO battery remains relatively stable during cycling (Figure 4h). These results indicate that the ZMO layer remains stable after cycling, thereby optimizing the electrochemical properties of the Zn anode (Figure S12).

**Figure 5:**
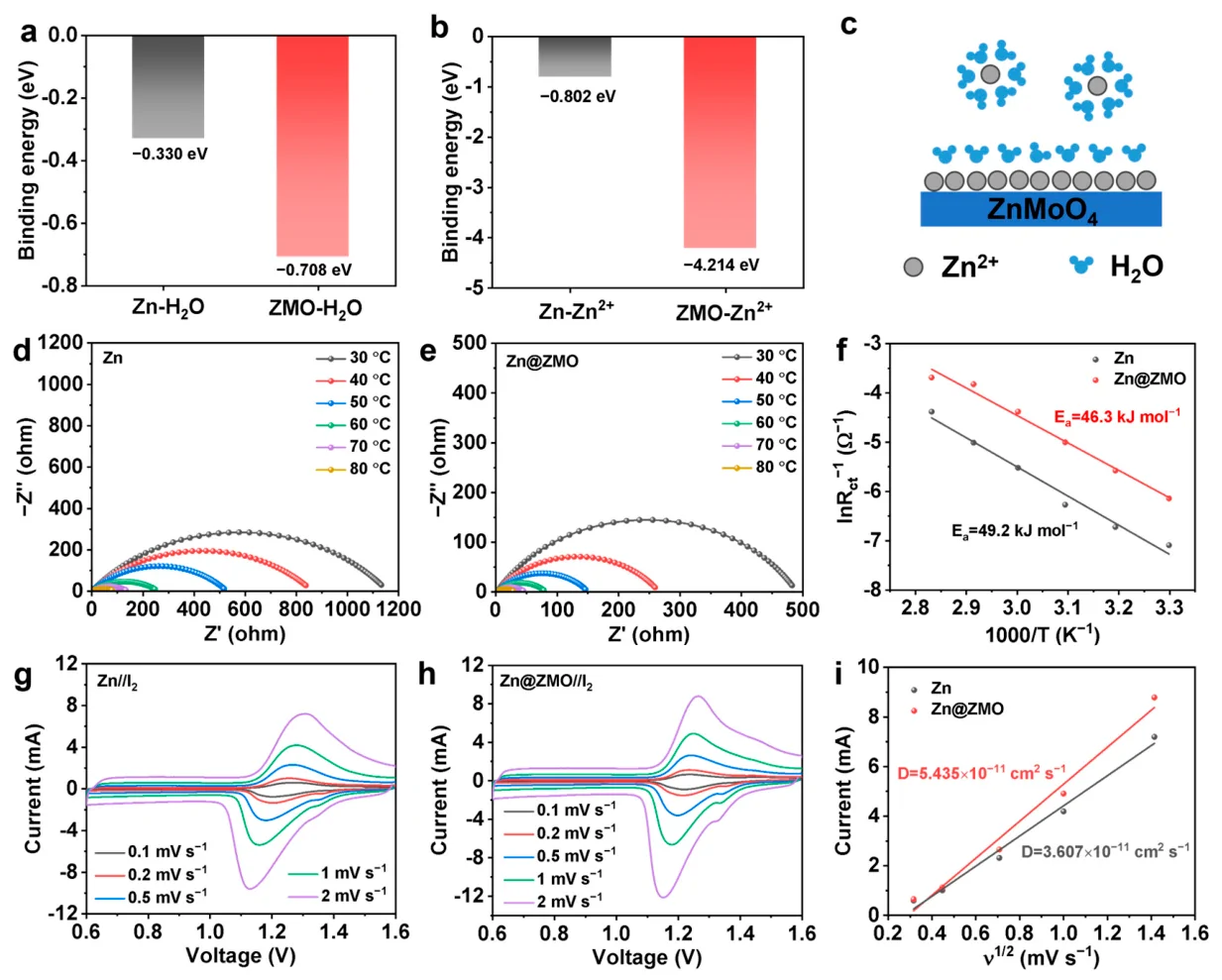
Adsorption energy of (**a**) the H_2_O molecule and (**b**) the Zn^2+^ ion on the Zn and Zn@ZMO surfaces. (**c**) Schematic diagram of desolvation at the ZMO interfaces. Nyquist curves of the (**d**) Zn//Zn and (**e**) Zn@ZMO//Zn@ZMO batteries from 30 to 80 °C. (**f**) Desolvation activation energies of the symmetrical batteries. CV curves of (**g**) Zn//I_2_ and (**h**) Zn@ZMO//I_2_ batteries. (**i**) The relationship linking the peak current (I_p_) to the square root of the scanning rate (ν^1/2^).

The ZMO protective layer not only shielded the Zn foil but also enhanced the transfer of Zn^2+^ ions. Density functional theory (DFT) was employed to compute the adsorption energies of both H_2_O molecules and Zn^2+^ onto the electrode surface. The higher the adsorption energy, the stronger the interfacial force, making it easier to absorb ions or molecules. As illustrated in Figure 5a–c, the adsorption energies of H_2_O and Zn^2+^ ions onto the Zn@ZMO surface were −0.708 eV and −4.214 eV, respectively, which were higher than those on the Zn anode (Zn-H_2_O: −0.330 eV, Zn-Zn^2+^: −0.802 eV). These findings demonstrated that the ZMO interface promoted the desolvation of Zn^2+^ ions. The variation in impedance with temperature for both Zn//Zn and Zn@ZMO//Zn@ZMO symmetrical batteries was also measured to determine the ionic conductivity. Fitting analyses of ionic conductivity demonstrated that the desolvation activation energy on the Zn@ZMO surface was 46.3 kJ mol^−1^, which is less than that observed for Zn (49.2 kJ mol^−1^) (Figure 5d–f). This further confirmed that the ZMO interfacial layer enhanced Zn^2+^ desolvation, thereby promoting faster Zn^2+^ transport. Cyclic voltammetry (CV) along with galvanostatic intermittent titration technique (GITT) analysis also revealed that the ZMO interfacial layer accelerated the diffusion rate of Zn^2+^ ions (Figure 5g,h and Figure S13). According to Figure 5i, the Zn@ZMO//I_2_ full battery exhibited a considerably higher Zn^2+^ ion diffusion coefficient (D_Zn_^2+^) (D = 5.435 × 10^−11^ cm^2^ s^−1^) compared to that of the Zn//I_2_ battery (D = 3.607 × 10^−11^ cm^2^ s^−1^). This improvement in electrochemical activity is attributable to the ability of the ZMO layer to promote Zn^2+^ desolvation.

**Figure 6:**
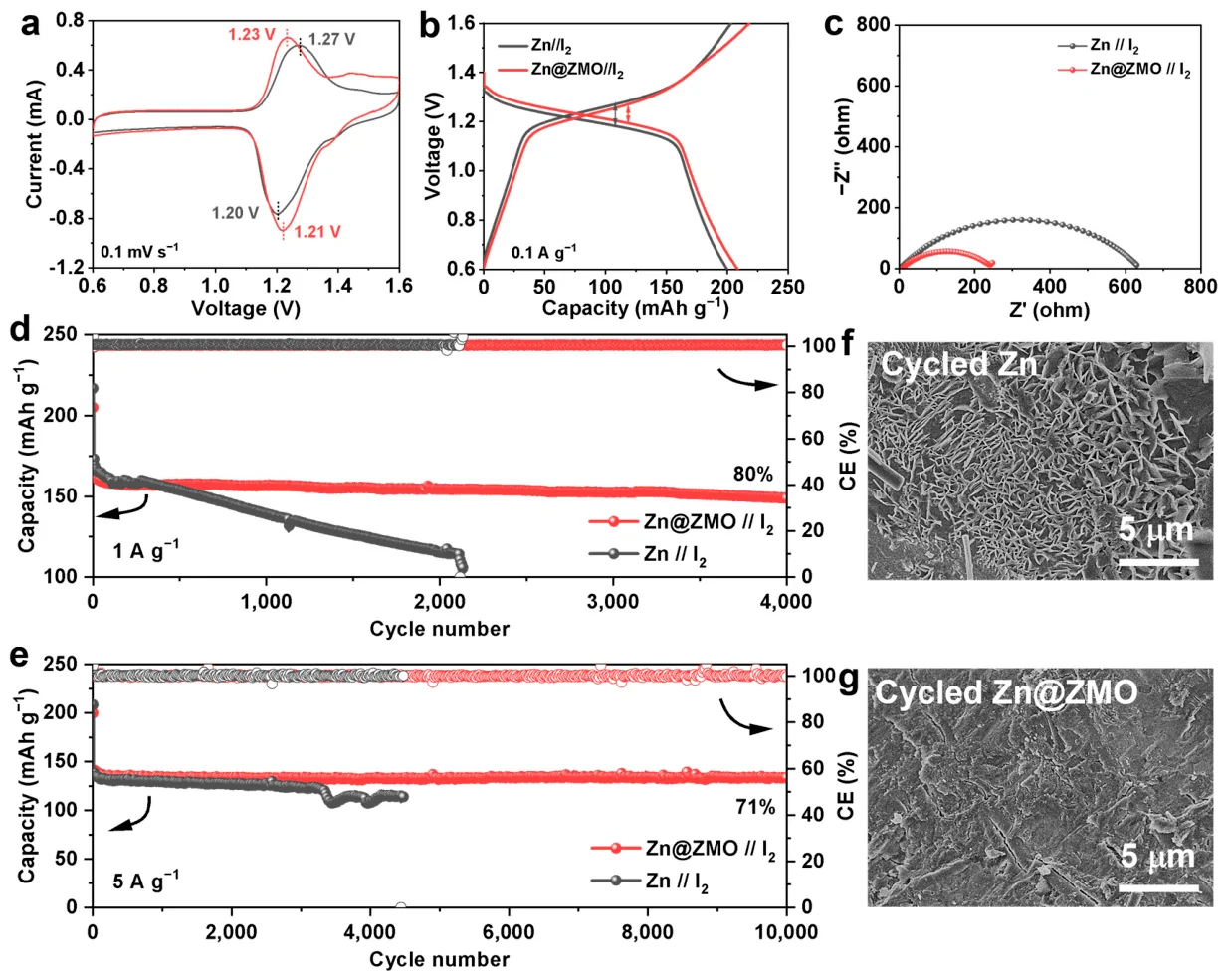
(**a**) CV curves of the Zn//I_2_ and Zn@ZMO//I_2_ batteries at 0.1 mV s^−1^. (**b**) Galvanostatic charge/discharge profiles of full batteries at 0.1 A g^−1^. (**c**) EIS of the full batteries. Cycling performance of the Zn//I_2_ and Zn@ZMO//I_2_ batteries at (**d**) 1 A g^−1^ and (**e**) 5 A g^−1^. Top-view SEM images of cycled (**f**) Zn and (**g**) Zn@ZMO electrodes from post-cycling full cells.

The practical utility of the ZMO protective layer was determined in a Zn@ZMO//I_2_ full cell. Both the CV curves of the Zn//I_2_ and Zn@ZMO//I_2_ full cells exhibited a reduction peak and an oxidation peak at 0.1 mV s^−1^. The Zn@ZMO//I_2_ cell (~0.02 V) delivered a lower voltage hysteresis than the Zn//I_2_ cell (~0.07 V), indicating reduced electrochemical polarization in the Zn@ZMO//I_2_ full cell (Figure 6a).

Additionally, the galvanostatic charge/discharge curves of the Zn@ZMO//I_2_ cell demonstrated lower polarization voltage compared to the Zn/I_2_ full cell (Figure 6b). Electrochemical polarization is linked to the charge-transfer resistance (R_ct_), which is manifested as a semicircle in the high- to medium-frequency portion of the Nyquist curve. Because the ZMO layer stabilizes the electrode interface, the Zn@ZMO//I_2_ battery showed a substantially reduced R_ct_ (241 Ω) compared with Zn//I_2_ (638 Ω). (Figure 6c). Furthermore, the Zn@ZMO//I_2_ cell retained a capacity of 149 mAh g^−1^ with 80% retention after 4000 cycles at 1 A g^−1^. Even at 5 A g^−1^, the Zn@ZMO//I_2_ battery maintained 133 mAh g^−1^ with 71% retention after 10,000 cycles, considerably outperforming the Zn//I_2_ battery (Figure 6d,e and Figure S14). After cycling characterization of the Zn and Zn@ZMO foils revealed distinct differences, the cycled Zn anode exhibited prominent sheet-like dendrites (Figure 6f), while the cycled Zn@ZMO anode maintained a relatively flat surface (Figure 6g). These outcomes indicated that the ZMO interlayer effectively protected the Zn foil and enhanced the electrochemical capability.

## 3. Conclusions

The ZMO protective interlayer, fabricated on Zn foil using a straightforward one-step immersion method, effectively mitigated corrosion, HERs, and Zn dendrite formation in aqueous electrolytes. The ZMO layer exhibited substantial adsorption energy for both H_2_O molecules and Zn^2+^ ions, facilitating the desolvation of Zn^2+^ ions, accelerating their migration, and enhancing their reversibility. Additionally, this protective layer reduced the polarization voltage between electrodes, promoted uniform Zn^2+^ ion deposition, and extended battery cycle life. As a result, the Zn@ZMO//Zn@ZMO battery achieved a cycle life of 1000 h at 5 mA cm^−2^ with 1 mAh cm^−2^ and remained stable even at 20 mA cm^−2^. This low-cost in situ immersion modification technology aligns with the industry’s core pursuit of process simplification, cost control, and performance improvement, and is considered one of the key paths to drive AZIBs from the laboratory to large-scale applications.

## Data Availability

Data are contained within the article. Further inquiries can be directed to the corresponding authors.

## Supplementary Materials

The following supporting information can be downloaded at https://www.mdpi.com/article/10.3390/mi17080982/s1. Figure S1. Schematic illustrations of hydrothermal and one-step immersion method; Figure S2. Schematic diagram of Zn@ZMO synthesis; Figure S3. XRD pattern of Zn foil soaking in the phosphomolybdate solution after 48 h; Figure S4. Raman of Zn foil, Zn@ZMO and Zn foil soaking in the phosphomolybdate solution after 48 h; Figure S5. Cycling stability of Zn anodes with different soaking times of 10, 30, and 60 min; Figure S6. Voltage-time profiles of the Zn//Zn and Zn@ZMO//Zn@ZMO symmetrical cells at (a,b) 1 mA cm^−2^ and (c) 5 mA cm^−2^; Figure S7. Polarization voltage of the Zn//Zn and Zn@ZMO//Zn@ZMO symmetrical cells at different current densities; Figure S8. LSV curves of pristine Zn and Zn@ZMO at 10 mV ^−1^ in 2 M Na_2_SO_4_ electrolyte; Figure S9. (a) XRD patterns and SEM images of (b) Zn and (c) Zn@ZMO deposited at 1 mA cm^−2^ for 5 h; Figure S10. (a) Optical photographs of Zn and Zn@ZMO electrodes soaking in electrolyte. (b) XRD pattern of soaked Zn and Zn@ZMO electrodes; Figure S11. XPS spectra of (a) Zn 2p, (b) Mo 3d, and (c) O 1s of Zn@ZMO after 50 cycles; Figure S12. EIS before and after 100 cycles for Zn//Zn and Zn@ZMO//Zn@ZMO cells; Figure S13. GITT potential profiles of Zn//I2 and Zn@ZMO//I2 full cells; Figure S14. Discharge/charge profiles of (a) Zn//I2 and (b) Zn@ZMO//I2 full cells at 1 A g^−1^; Table S1: Electrochemical performances of the modified Zn anodes in recent years [34,35,36,37,38]. Movie S1. the In situ low-resolution optical microscope of deposited Zn at 5 mA cm^−2^ onto Zn; Movie S2. the In situ low-resolution optical microscope of deposited Zn at 5 mA cm^−2^ onto Zn@ZMO.

## References

[1] Deng Q., Zhang Q., Chu Y., Xu Y., You S., Huang K., Yang C., Lu J. (2024). Understanding improved stability of Co-free Ni-rich single crystal cathode materials by combined bulk and surface modifications. Mater. Today.

[2] Xie C., Wang C., Xu Y., Li T., Fu Q., Li X. (2024). Reversible multielectron transfer I^−^/IO_3_^−^ cathode enabled by a hetero-halogen electrolyte for high-energy-density aqueous batteries. Nat. Energy.

[3] Jiang L., Han S., Hu Y.-C., Yang Y., Lu Y., Lu Y.-C., Zhao J., Chen L., Hu Y.-S. (2024). Rational design of anti-freezing electrolytes for extremely low-temperature aqueous batteries. Nat. Energy.

[4] Wan H., Xu J., Wang C. (2024). Designing electrolytes and interphases for high-energy lithium batteries. Nat. Rev. Chem..

[5] Dai Y., Lu R., Zhang C., Li J., Yuan Y., Mao Y., Ye C., Cai Z., Zhu J., Li J. (2024). Zn^2+^-mediated catalysis for fast-charging aqueous Zn-ion batteries. Nat. Catal..

[6] Yang X., Zhao Y., Lv S., Zhong L., Yue C., Zhan S., Zhao L., Wang C., Li X., Liu X. (2024). Anion-promoted CB[6] macromolecule dissolution for stable Zn-ion batteries. Energy Environ. Sci..

[7] Zhang Z., Zhang Y., Ye M., Wen Z., Tang Y., Liu X., Li C.C. (2023). Lithium Bis(oxalate)borate Additive for Self-repairing Zincophilic Solid Electrolyte Interphases towards Ultrahigh-rate and Ultra-stable Zinc Anodes. Angew. Chem. Int. Ed..

[8] Cheng Z., Wang K., Fu J., Mo F., Lu P., Gao J., Ho D., Li B., Hu H. (2024). Texture Exposure of Unconventional (101)Zn Facet: Enabling Dendrite-Free Zn Deposition on Metallic Zinc Anodes. Adv. Energy Mater..

[9] Chen Y., Yin J., Zhang Y., Lyu F., Qin B., Zhou J., Liu J.-H., Long Y.-C., Mao Z., Miao M. (2024). Coupling High Hardness and Zn Affinity in Amorphous–Crystalline Diamond for Stable Zn Metal Anodes. ACS Nano.

[10] Yan C., Lv C., Wang L., Cui W., Zhang L., Dinh K.N., Tan H., Wu C., Wu T., Ren Y. (2020). Architecting a Stable High-Energy Aqueous Al-Ion Battery. J. Am. Chem. Soc..

[11] Li R., Yu J., Chen F., Su Y., Chan K.C., Xu Z.-L. (2023). High-Power and Ultrastable Aqueous Calcium-Ion Batteries Enabled by Small Organic Molecular Crystal Anodes. Adv. Funct. Mater..

[12] Wei T., Ren Y., Wang Y., Mo L.E., Li Z., Zhang H., Hu L., Cao G. (2023). Addition of Dioxane in Electrolyte Promotes (002)-Textured Zinc Growth and Suppressed Side Reactions in Zinc-Ion Batteries. ACS Nano.

[13] Hu Z., Wang X., Du W., Zhang Z., Tang Y., Ye M., Zhang Y., Liu X., Wen Z., Li C.C. (2023). Crowding Effect-Induced Zinc-Enriched/Water-Lean Polymer Interfacial Layer Toward Practical Zn-Iodine Batteries. ACS Nano.

[14] Shi M., Lei C., Wang H., Jiang P., Xu C., Yang W., He X., Liang X. (2024). Molecule Engineering of Sugar Derivatives as Electrolyte Additives for Deep-Reversible Zn Metal Anode. Angew. Chem. Int. Ed..

[15] Liu Z., Wang R., Ma Q., Wan J., Zhang S., Zhang L., Li H., Luo Q., Wu J., Zhou T. (2024). A Dual-Functional Organic Electrolyte Additive with Regulating Suitable Overpotential for Building Highly Reversible Aqueous Zinc Ion Batteries. Adv. Funct. Mater..

[16] You S., Deng Q., Wang Z., Chu Y., Xu Y., Lu J., Yang C. (2024). Achieving Highly Stable Zn Metal Anodes at Low Temperature via Regulating Electrolyte Solvation Structure. Adv. Mater..

[17] Wan J., Wang R., Liu Z., Zhang L., Liang F., Zhou T., Zhang S., Zhang L., Lu Q., Zhang C. (2023). A Double-Functional Additive Containing Nucleophilic Groups for High-Performance Zn-Ion Batteries. ACS Nano.

[18] Nie C., Wang G., Wang D., Wang M., Gao X., Bai Z., Wang N., Yang J., Xing Z., Dou S. (2023). Recent Progress on Zn Anodes for Advanced Aqueous Zinc-Ion Batteries. Adv. Energy Mater..

[19] Lu W., Zhang C., Zhang H., Li X. (2021). Anode for Zinc-Based Batteries: Challenges, Strategies, and Prospects. ACS Energy Lett..

[20] Qiu H., Du X., Zhao J., Wang Y., Ju J., Chen Z., Hu Z., Yan D., Zhou X., Cui G. (2019). Zinc anode-compatible in-situ solid electrolyte interphase via cation solvation modulation. Nat. Commun..

[21] Chen J., Xiong J., Ye M., Wen Z., Zhang Y., Tang Y., Liu X., Li C.C. (2024). Suppression of Hydrogen Evolution Reaction by Modulating the Surface Redox Potential Toward Long-Life Zinc Metal Anodes. Adv. Funct. Mater..

[22] Yi C., Jiao L., Wang J., Ma Y., Bai H., Liu Q., Wang S., Xin W., Lei Y., Zhang T. (2024). Multi-Scale Functionally Designed ZnWO4 Artificial Interphase for Ultra-Stable Aqueous Zn Metal Anodes Under High Current Rates. Adv. Funct. Mater..

[23] Kang L., Cui M., Jiang F., Gao Y., Luo H., Liu J., Liang W., Zhi C. (2018). Nanoporous CaCO_3_ Coatings Enabled Uniform Zn Stripping/Plating for Long-Life Zinc Rechargeable Aqueous Batteries. Adv. Energy Mater..

[24] Song T.-B., Ma Q.-L., Zhang X.-R., Ni J.-W., He T.-L., Xiong H.-M. (2023). Zn anode surface engineering for stable zinc-ion batteries: Carbon dots incorporated mesoporous TiO_2_ as a coating layer. Chem. Eng. J..

[25] Wei B., Zheng J., Abhishek, Liu X., Wu J., Qi Z., Hou Z., Wang R., Ma J., Gandi A.N. (2024). Design Principle of Insulating Surface Protective Layers for Metallic Zn Anodes: A Case Study of ZrO_2_. Adv. Energy Mater..

[26] Zhang S., Ye M., Zhang Y., Tang Y., Liu X., Li C.C. (2023). Regulation of Ionic Distribution and Desolvation Activation Energy Enabled by In Situ Zinc Phosphate Protective Layer toward Highly Reversible Zinc Metal Anodes. Adv. Funct. Mater..

[27] Liu X., Yang F., Xu W., Zeng Y., He J., Lu X. (2020). Zeolitic Imidazolate Frameworks as Zn^2+^ Modulation Layers to Enable Dendrite-Free Zn Anodes. Adv. Sci..

[28] Cao P., Zhou X., Wei A., Meng Q., Ye H., Liu W., Tang J., Yang J. (2021). Fast-Charging and Ultrahigh-Capacity Zinc Metal Anode for High-Performance Aqueous Zinc-Ion Batteries. Adv. Funct. Mater..

[29] You S., Liu D.-S., Ye M., Zhang Y., Tang Y., Liu X., Li C.C. (2023). Graphite intercalation compounds (GICs) based multi-functional interface layer toward highly reversible Zn metal anodes. Chem. Eng. J..

[30] Chen A., Zhao C., Guo Z., Lu X., Liu N., Zhang Y., Fan L., Zhang N. (2022). Fast-growing multifunctional ZnMoO_4_ protection layer enable dendrite-free and hydrogen-suppressed Zn anode. Energy Storage Mater..

[31] Ma L., Schroeder M.A., Borodin O., Pollard T.P., Ding M.S., Wang C., Xu K. (2020). Realizing high zinc reversibility in rechargeable batteries. Nat. Energy.

[32] Chen C., Xue L., Li G., Chen X., Han Y., Mi L., Chen Y. (2026). Long-Chain Molecule Reconstruction of Novel Solvation Structure to Stabilize Zinc Metal Anode. Adv. Sci..

[33] Deng Q., You S., Min W., Xu Y., Lin W., Lu J., Yang C. (2024). Polymer Molecules Adsorption-Induced Zincophilic-Hydrophobic Protective Layer Enables Highly Stable Zn Metal Anodes. Adv. Mater..

[34] Xu P., Wang C., Zhao B., Zhou Y., Cheng H. (2021). An interfacial coating with high corrosion resistance based on halloysite nanotubes for anode protection of zinc-ion batteries. J. Colloid Interface Sci..

[35] Zhang N., Huang S., Yuan Z., Zhu J., Zhao Z., Niu Z. (2021). Direct self-assembly of MXene on Zn anodes for dendrite-free aqueous zinc-ion batteries. Angew. Chem. Int. Ed..

[36] Liu M., Cai J., Ao H., Hou Z., Zhu Y., Qian Y. (2020). NaTi2(PO4)3 solid-state electrolyte protection layer on Zn metal anode for superior long-life aqueous zinc-ion batteries. Adv. Funct. Mater..

[37] Li Z., Wu L., Dong S., Xu T., Li S., An Y., Jiang J., Zhang X. (2021). Pencil drawing stable interface for reversible and durable aqueous zinc-ion batteries. Adv. Funct. Mater..

[38] Huang S., Tang R., Liu X., Zhang Y., Tang Y., Wen Z., Ye M., Yang Y., Li C.C. (2024). Ion–dipole interaction motivated Zn2+ pump and anion repulsion interface enable ultrahigh-rate Zn metal anodes. Energy Environ. Sci..

