# Supplementary material for: Rational In Situ Fabrication of ZnMoO4 Shielding Layers to Mitigate Zinc Degradation and Extend Battery Lifespan
Source: Micromachines (Basel). 2026 Aug 20;17(8):982. doi: 10.3390/mi17080982 (PMC13515221; doi:10.3390/mi17080982)
Supplement: Supplementary file 1 [file micromachines-17-00982-s001.zip › micromachines-4469625-supplementary.pdf]

Supporting Information

Rational In Situ Fabrication of $ZnMoO_4$ Shielding Layers to Mitigate Zinc Degradation and Extend Battery Lifespan

Xiaodong Zhang [1,†], Yan Zhang [2,†], Yingbin Liu [1], Kai Li [1,*] and Changdong Chen [1,*]

1 School of Materials Science and Engineering, Pingdingshan University, Pingdingshan 467000, China
2 State Key Laboratory of Space Power-Sources, Harbin Institute of Technology, Harbin 150001, China; zhangyhit@hit.edu.cn
* Correspondence: 20250013@pdsu.edu.cn (K.L.); lmccd5613@163.com (C.C.)
† These authors contributed equally to this work.

Experimental section

Preparation of Zn@ZMO

A total of 200 μL of a 0.1 M phosphomolybdate solution was applied to the surface of Zn foil (13 mm discs, 100 μm thick) and left to stand for 30 min. The excess solution was then removed by washing with deionized water, followed by drying at 80 °C for 3 h to obtain Zn@ZMO.

Material Characterizations

Field-emission scanning electron microscopy (FESEM, Hitachi SU8010) was used to examine the microstructures of the samples. X-ray powder diffraction (XRD, Bruker D8, Cu Kα) was applied to characterize the crystal structures. Additionally, X-ray photoelectron spectroscopy (XPS, Escalab Xi+) served to analyze the chemical states on the anode surface. The roughness of cycled anodes was observed using a confocal laser scanning microscope (CLSM, Olympus OLS4100). The contact angles of the anodes with the electrolyte were measured using a DSA25 instrument (Germany Krüss).

Electrochemical Measurements

For the Zn-I_2 full cell, iodine powder and activated carbon were homogeneously mixed in a 1:1 mass ratio as the active materials. The active materials (80 wt%), carboxymethyl cellulose (CMC; 10 wt%), and acetylene black (10 wt%) were combined with deionized water to form a slurry, which was then coated onto carbon paper to prepare the cathode. The mass loading of the I_2 cathode was approximately 3–4 mg cm^{-2}. Zn or Zn@ZMO served as the counter electrode, with 2 M $ZnSO_4$ (ZSO) in deionized water as the electrolyte and glass fiber as the separator. Galvanostatic discharge/charge (GDC) tests were conducted on a LAND CT2001 battery testing system at 27 °C, with cycling at different current densities within a voltage range of 0.6–1.6 V. Electrochemical impedance spectroscopy and CV tests were performed using a CHI660e electrochemical workstation. For GITT measurements, the current pulse duration was set to 10 min at 0.1 A g^{-1}, with an interval time of 1 h.

The diffusion coefficient of Zn^{2+} ions, determined from CV, was calculated using the following equation:

$$I_p = (2.69 \times 10^5) n^{3/2} A D^{1/2} C v^{1/2}$$

where I_p denotes the peak current (A), n denotes the number of reaction electrons, A denotes the contact area between the electrolyte and electrode (cm^2), D denotes the diffusion coefficient Zn^{2+} ions ($cm^2\ s^{-1}$), C denotes the concentration of Zn^{2+} ions in the electrode (mol cm^{-3}), and v denotes the scan rate (V s^{-1}).

DFT Simulation Method

Density functional theory (DFT) computations were conducted with the projector-augmented wave (PAW) approach within the Vienna ab initio simulation package (VASP). To evaluate the adsorption energy, the generalized gradient approximation (GGA) in the form of the Perdew–Burke–Ernzerhof (PBE) functional was applied. The energy cutoff was set at 520 eV, with a Gamma-centered 1×1×1 k-points mesh applied to all calculations. Structural relaxation was performed until the residual forces on all atoms fell below -0.03 eV $Å^{-1}$ and the total energy converged to within 1×10^{-5} eV. The binding energy (E_b) between the Zn/$ZnMoO_4$ matrix and the molecule was then computed according to the following equation:

$$E_{absorb\ energy} = E_{total} - E_{matrix} - E_{molecuar}$$

where $E_{molecuar}$ denotes the energy of electrolyte molecule, and E_{matrix} denotes the energy of Zn matrix or $ZnMoO_4$ matrix; E_{Total} denotes the total energy of molecule adsorbed on Zn matrix, and $E_{absorb\ energy}$ denotes the absorb energy (eV).

COMSOL Simulation Method

COMSOL Multiphysics software was employed to perform finite element analysis (FEA) simulations. The FEA model was defined by the law of mass conservation and the electroneutrality assumption of related ions, without considering possible side reactions. The domain was created with the corresponding dimensions as presented in the figures, with the top line selected as the positive electrode surface and the bottom rectangle as the anode domain. The middle region represented the area where electrolyte and ion flow occurred. Two different anode domains were established to characterize the effect of anode materials on Zn^{2+} ion transport and migration.

The size of the computational domain was set to 6 μm (width) × 6 μm (height) to simplify the calculation. The simulation steps included current distribution initialization and a transient step. A zero electric potential (0 V) was imposed on all negative electrode surfaces, whereas the positive electrodes were subjected to an average current density of 100 mA cm^{-2}. The conductivity of the bulk electrolyte was set to 1×10^{-3} S cm^{-1} and the starting Zn^{2+} ion concentration in the electrolyte was 2000 mol m^{-3}. For the purpose of initial electric field simulation, minor protrusions were introduced on the material surface to account for the influence of early Zn dendrite nucleation points.

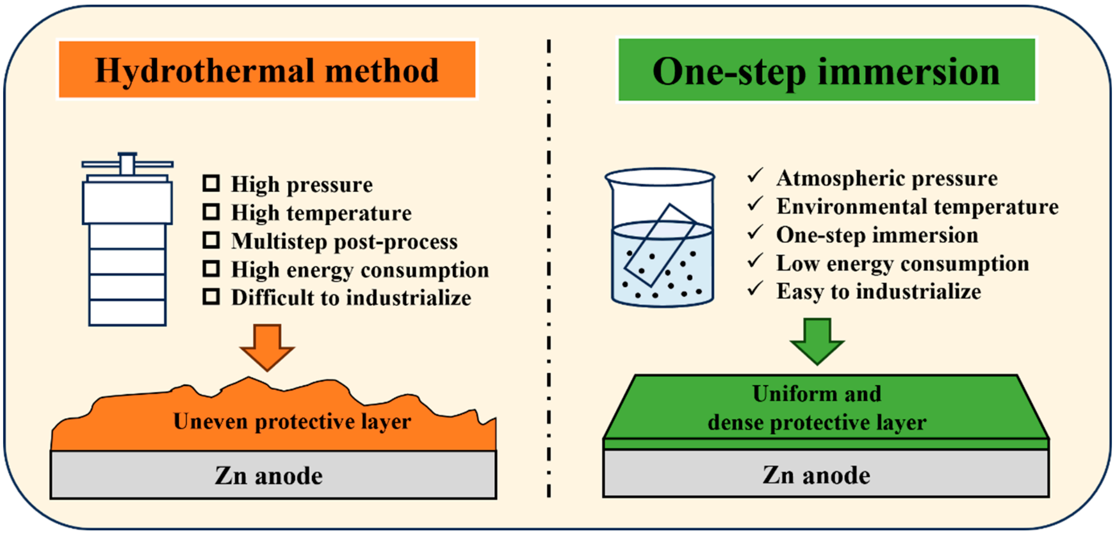

Figure S1. Schematic illustrations of hydrothermal and one-step immersion method.

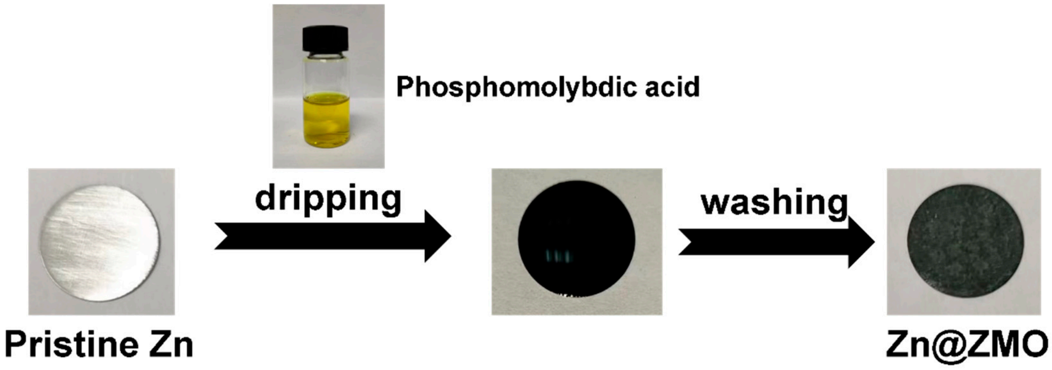

Figure S2. Schematic diagram of Zn@ZMO synthesis.

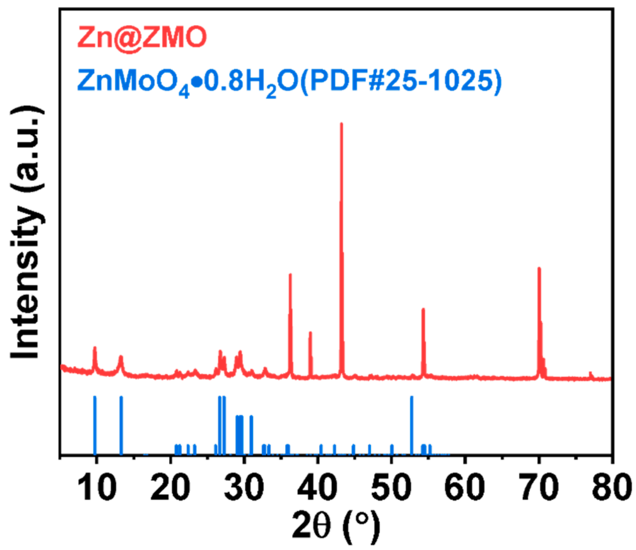

Figure S3. XRD pattern of Zn foil soaking in the phosphomolybdate solution after 48 h.

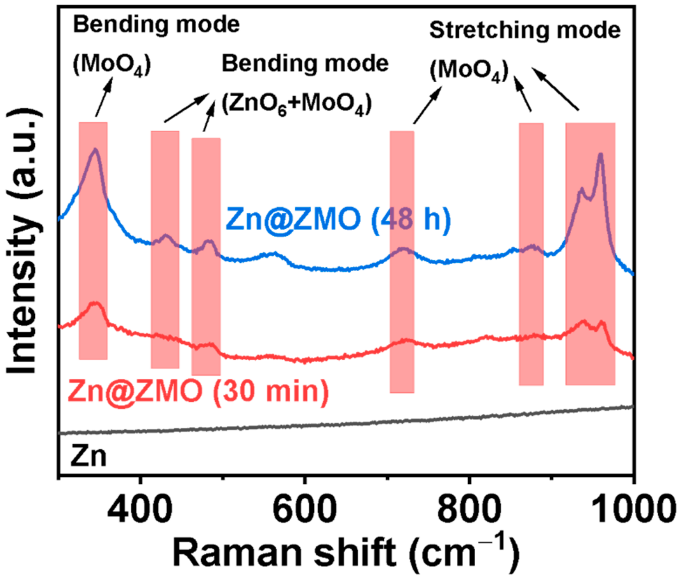

Figure S4. Raman of Zn foil, Zn@ZMO and Zn foil soaking in the phosphomolybdate solution after 48 h.

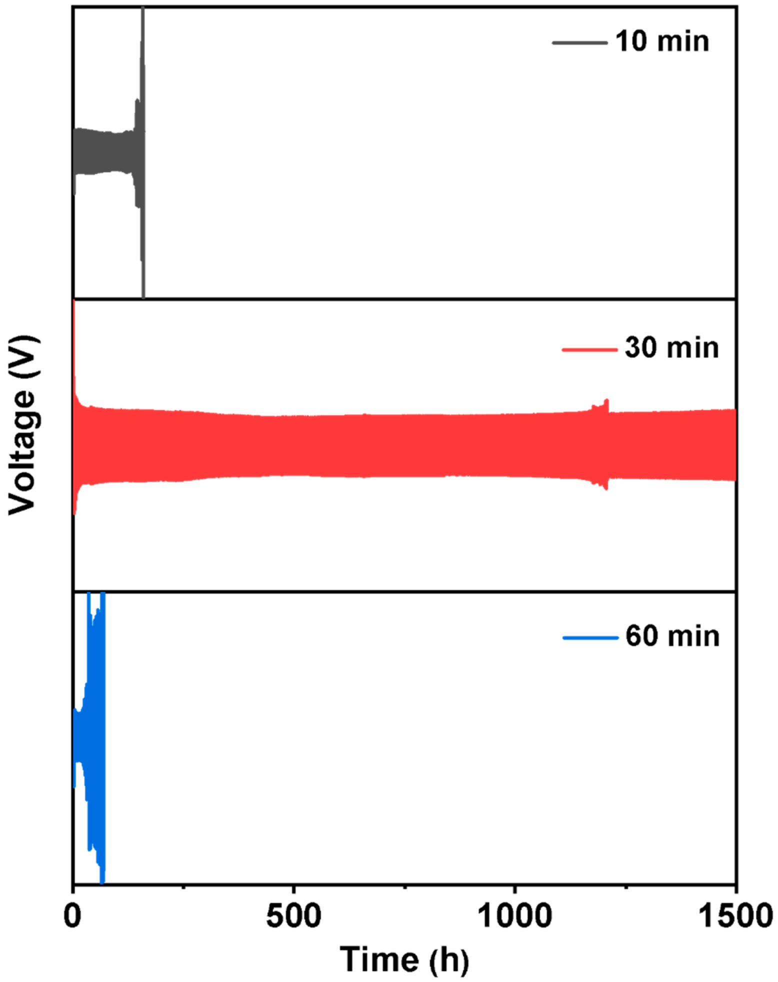

Figure S5. Cycling stability of Zn anodes with different soaking times of 10, 30, and 60 min.

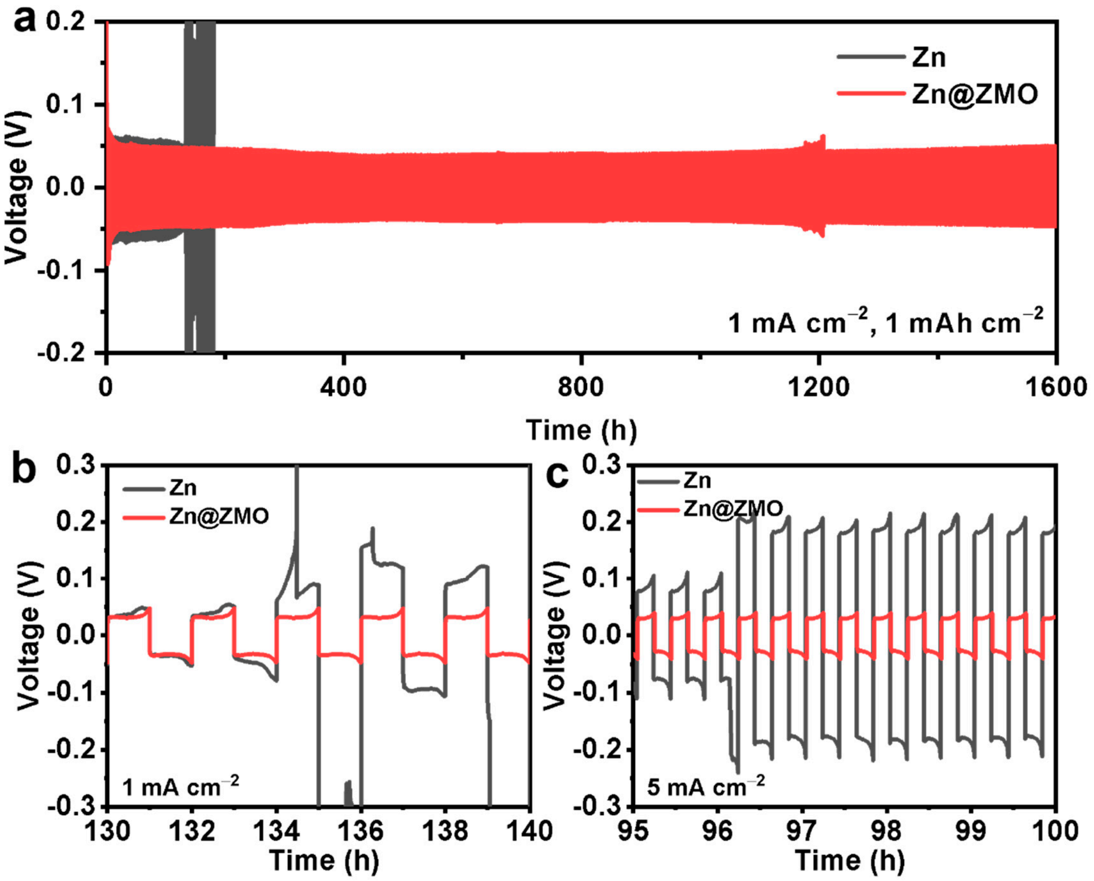

Figure S6. Voltage-time profiles of the Zn//Zn and Zn@ZMO//Zn@ZMO symmetrical cells at (a, b) 1 mA cm^{-2} and (c) 5 mA cm^{-2}.

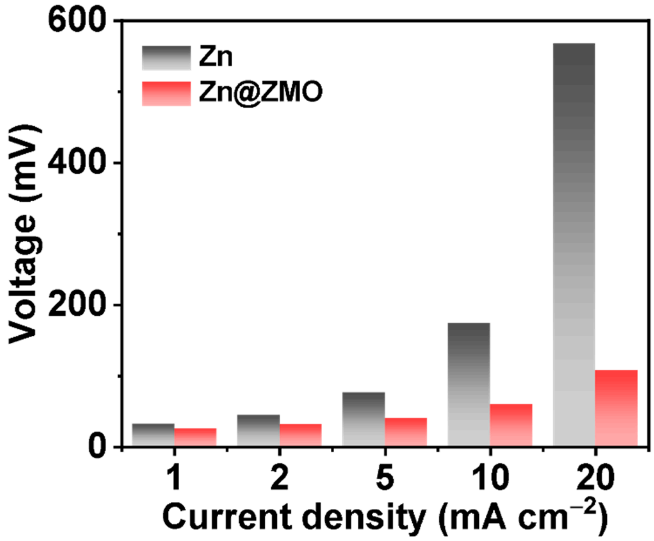

Figure S7. Polarization voltage of the Zn//Zn and Zn@ZMO//Zn@ZMO symmetrical cells at different current densities.

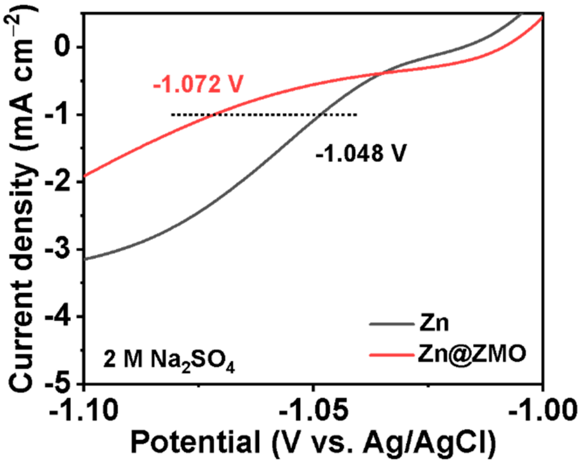

Figure S8. LSV curves of pristine Zn and Zn@ZMO at 10 mV s^{-1} in 2 M Na_2SO_4 electrolyte.

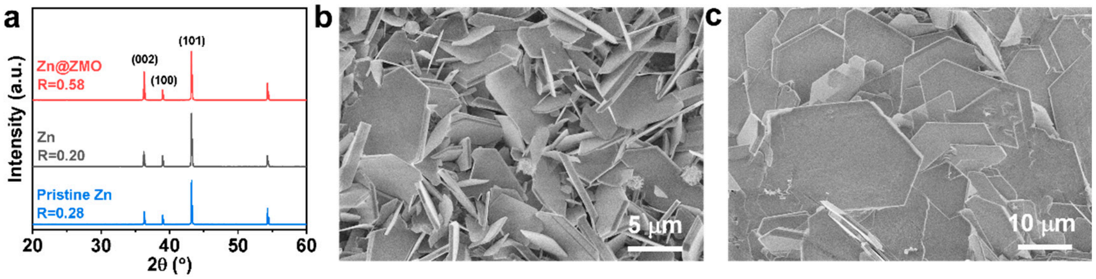

Figure S9. (a) XRD patterns and SEM images of (b) Zn and (c) Zn@ZMO deposited at 1 mA cm^{-2} for 5 h.

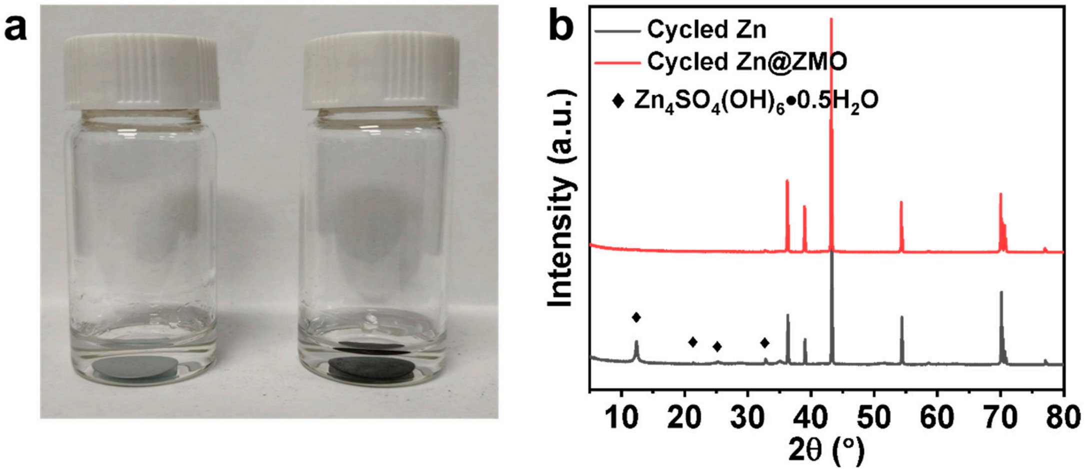

Figure S10. (a) Optical photographs of Zn and Zn@ZMO electrodes soaking in electrolyte. (b) XRD pattern of soaked Zn and Zn@ZMO electrodes.

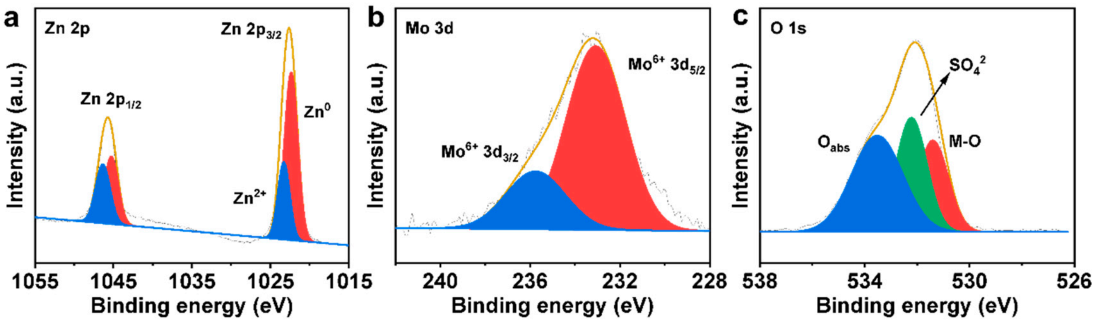

Figure S11. XPS spectra of (a) Zn 2p, (b) Mo 3d, and (c) O 1s of Zn@ZMO after 50 cycles.

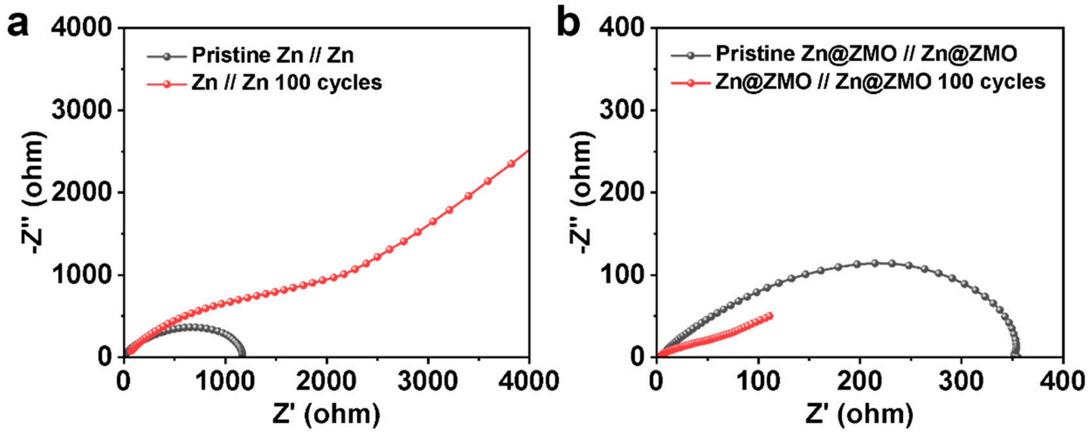

Figure S12. EIS before and after 100 cycles for Zn // Zn and Zn@ZMO // Zn@ZMO cells.

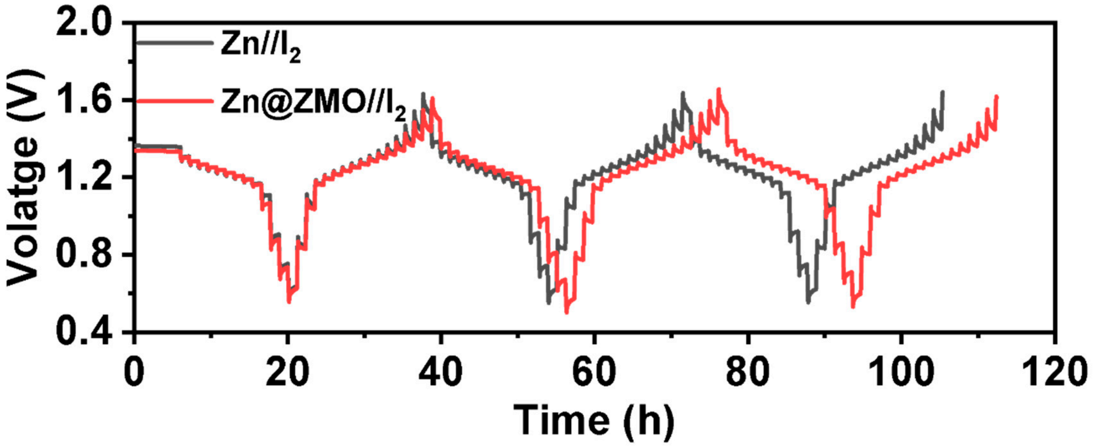

Figure S13. GITT potential profiles of $Zn//I_2$ and $Zn@ZMO//I_2$ full cells.

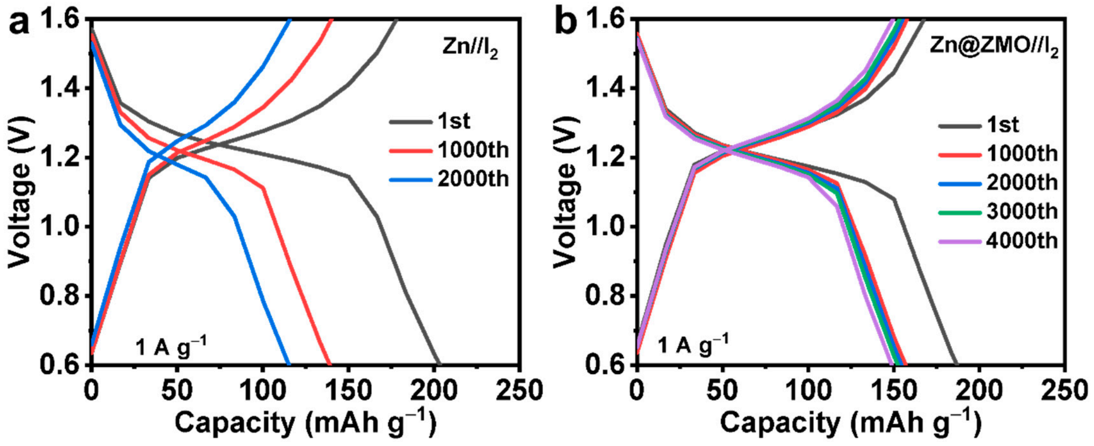

Figure S14. Discharge/charge profiles of (a) Zn//I_2 and (b) Zn@ZMO//I_2 full cells at 1 A g^{-1}.

Table S1. Electrochemical performances of the modified Zn anodes in recent years.

**Anode**	**Electrolyte**	**Voltage hysteresis (mV)**	**Current density (mA cm^{-2})**	**Areal capacity (mAh cm^{-2})**	**Ref.**
HNTs-Zn	2 M $ZnSO_4$	25/30/40/60	0.25/0.5/1/2	0.5/0.1/0.2/0.4	S1
MXene-coated Zn	2 M $ZnSO_4$	48/54/80/112	0.5/1/3/5	-	S2
Zn@ZIF-8	2 M $ZnSO_4$	15/20/35/40/50	0.5/1/2/3/5	0.5/0.5/0.5/0.5/0.5	S3
Zn@ZnP	2 M $ZnSO_4$	25/50/70/80/100	0.2/1/2/5/10	-	S4
Zn@MGs	3 M $Zn(CF_3SO_3)_2$	25/30/70/100	1/2/5/10	1/1/1/1/1/1	S5
NTP@Zn	2 M $ZnSO_4$	24/25/30/35/50	0.2/0.4/1/2/5	0.2/0.4/1/2/5	S6
Graphite-coated Zn	2 M $ZnSO_4$	27.3/28/30/35/50/73	0.1/0.2/0.4/0.8/1.0/1.5	0.1/0.2/0.4/0.8/1.0/1.5	S7
Zn@PAPF	2 M $ZnSO_4$	26/33/45/68	1/2/5/10	1/1/1/1/1	S8
**Zn@ZMO**	**2 M $ZnSO_4$**	**25.6/31.6/40.6/60.1/107.4**	**1/2/5/10/20**	**1/1/1/1/1**	**This work**

Notes: Halloysite nanotubes (HNTs), $NaTi_2(PO_4)_3$ (NTP), Zeolitic imidazolate framework-8 (ZIF-8), Graphite intercalation compounds of molybdenum chloride (MGs), Perfluoropentanoic acid (PFPA).

References

[S1] P. Xu, C. Wang, B. Zhao, Y. Zhou, H. Cheng, An interfacial coating with high corrosion resistance based on halloysite nanotubes for anode protection of zinc-ion batteries, J. Colloid Interface Sci. 602 (2021) 859-867.

[S2] N. Zhang, S. Huang, Z. Yuan, J. Zhu, Z. Zhao, Z. Niu, Direct self-assembly of MXene on Zn anodes for dendrite-free aqueous zinc-ion batteries, Angew. Chem., Int. Ed. 60 (2021) 2861-2865.

[S3] X. Liu, F. Yang, W. Xu, Y. Zeng, J. He, X. Lu, Zeolitic imidazolate frameworks as Zn^{2+} modulation layers to enable dendrite-free Zn anodes, Adv. Sci. 7 (2020) 2002173.

[S4] P. Cao, X. Zhou, A. Wei, Q. Meng, H. Ye, W. Liu, J. Tang, J. Yang, Fast-charging and ultrahigh-capacity zinc metal anode for high-performance aqueous zinc-ion batteries, Adv. Funct. Mater. 31 (2021) 2100398.

[S5] S. You, D.-S. Liu, M. Ye, Y. Zhang, Y. Tang, X. Liu, C. Chao Li, Graphite intercalation compounds (GICs) based multi-functional interface layer toward highly reversible Zn metal anodes, Chem. Eng. J. 454 (2023) 139907.

[S6] M. Liu, J. Cai, H. Ao, Z. Hou, Y. Zhu, Y. Qian, $NaTi_2(PO_4)_3$ solid-state electrolyte protection layer on Zn metal anode for superior long-life aqueous zinc-ion batteries, Adv. Funct. Mater. 30 (2020) 2004885.

[S7] Z. Li, L. Wu, S. Dong, T. Xu, S. Li, Y. An, J. Jiang, X. Zhang, Pencil drawing stable interface for reversible and durable aqueous zinc-ion batteries, Adv. Funct. Mater. 31 (2021) 2006495.

[S8] S. Huang, R. Tang, X. Liu, Y. Zhang, Y. Tang, Z. Wen, M. Ye, Y. Yang, C.C. Li, Ion–dipole interaction motivated Zn^{2+} pump and anion repulsion interface enable ultrahigh-rate Zn metal anodes, Energy Environ. Sci. 17 (2024) 591-601.
